# Keeping up Appearances

**Published:** 1877-04

**Authors:** Ben. H. Pratt


### THE BISTOURY.

Vol. XIII. ELMIRA, N. Y., APRIL, 1877. No. 1.

### Contributions.

### KEEPING UP APPEARANCES.

BEN. H. PRATT.

Howsoever widely philanthropy thinks it has extended its beneficent arms, it should be reminded that it has failed to embrace at least one truly pitiable class of society; howsoever closely it believes it has scanned the miseries of our race, be it known that it has utterly overlooked an element in every community which cannot for many more generations bear up under the burdens to which it is being subjected. Enduring, and yet uncomplaining—suffering, and yet silent—patient, and yet hopeless—the married women of the middle classes in this country are slowly but surely degenerating, physically and mentally. The fact is notorious—the cause patent—the remedy easy; and yet, amid all the isms and ologies ameliorative, there is not one to champion the cause of those whom society has consigned to a serfdom as galling as any to which an inferior race was ever subjected by a superior—an oppression as surely extinctive as absolutism could invent.

*The fact.* Let any one of an observing turn of mind look about his own neighborhood for a well woman of the class alluded to. Let him note the pallor of this one—the distressed face of that one—the emaciation of another one. Let him for one month devote a reasonable share of observation to the langor, the ennui, the nervousness, the anxiety, the indisposition, the aches, the general tendency to invalidism that characterize the entire class of women who personally have the care of households. Select the healthiest of these you may—one whom you meet occasionally and who seems the embodiment of healthful vigor and perennial cheerfulness—acquaint yourself with her everyday life and learn how far from well this woman is. There are those who are the gayest in society that are the saddest at home—whose laugh is the seeming result of absolute joyousness, when it is but the hysterical relaxation from a pent up misery—to whom surcease of care is so great a novelty that it intoxicates, and gilds melancholy with a fictitious ecstasy for the nonce. There is no class of beings so widely different in their seeming and being as these. From the wife who drags herself from room to room, and heroically fights, inch by inch, the insidious disease that is sapping her vitality day by day, to her who, by reason of greater physical strength, bears the mental strain that management imposes, and stands the wear and tear consequent upon the work she cannot endure to leave undone, until nature yields all at once, and the doctor comes; between these two extremes lie every possible degree of unhealth and misery that can be conceived of, and not one in a hundred of those who manage households without unlimited means, is a well woman. Don't believe it? Get the confidence of husbands, or what is better, ask the doctors.

*The cause.* Few are content to conform their homes to their means. Society has become an arbitrary autocrat and demands of her subjects that each must endeavor to live a little better than he can afford. Society, while it recognizes differences in incomes, tolerates with bad grace any apparent difference in outgoes. The family with fifteen hundred or two thousand a year must live in as large a house as that family which enjoys a revenue of three or five thousand. Its rooms must be as many and as large; the furniture as good and as abundant; the carpets as costly and as heavy; the table as elegant and the cuisine as various; the companies as large and as frequent; else caste is lost. The smaller income may go thus far and keep pace with the larger, and cheat no one. But it ends here. This is the pomp and show part. The trouble begins with the management. The richer man has his cook and his chambermaid, and his maid of all work, and his serving man. The mistress is relieved of all labor, and delegates her cares to a subordinate. The poorer man, with the same establishment to keep up, has a maid of all work, and only this. Only the heaviest of the drudgery is taken from the shoulders of the mistress, and there is none to whom the household cares and responsibilities may be delegated. The former's life is one of comparative freedom from anxiety, the latter's is an unceasing round of labor and vexation and silent repinings, but she prefers to fret her life away rather than to exhibit to the world the fact which ninety-nine out of every hundred already know, to-wit: that she's only half as rich as her neighbor. Modern houses, with a number of useless rooms, containing much useless furniture, entail much work. If one has not the means of hiring the work done, one must do it one's self. Thus, merely for appearance sake, women will toil and contrive and fret—will lift, and reach, and sweep, and dust, and rub, and polish, perspire and take cold, breathe dust and cough—in a word, wear themselves out utterly, and with no better excuse for it than that they want to seem just as genteel as other people, defining gentility as that condition by which a thousand dollar family can keep up a two thousand dollar appearance. Perhaps sympathy for such is thrown away, inasmuch as their sufferings are self imposed; and an appeal in their behalf may be subjected to the same criticism that applies to people who don't mind their own business. But such argument would as well apply to the home missionary who visits the haunts of vice and crime with a view to elevating his race above those demoralizing influences, notwithstanding the results are strictly within the class of self-imposed wrongs. Society as truly needs ministering unto in respect to its social relations as it does in respect to its moral obligations. It should be taught that the end and aim of life is not social position; that a two thousand dollar family can't live a three thousand dollar life without cheating somebody out of a thousand dollars or its equivalent; that it's likelier to be the equivalent than the dollars, and the equivalent is, first, struggle, then discouragement, then chronic unhappiness, then invalidism, when it may be too late to repent.

*The remedy.* The sooner that people arrive at a philosophical way of thinking about the very uneven way this world's favors are distributed; as soon as folks will become reconciled to the fact that he who deserves the most very seldom gets it, but that it's all a matter of mystery that will not be explained; that all should live in such a way as to make what they have, and not what somebody else has, the standard of their mode of living; that happiness does not consist in pretending to be what one is not, but in doing the best with what one has; that misery is the result of strained effort to secure the unattainable, and happiness the contented settling down into that sphere that one seems to be destined for; then society will become naturally and not artificially classified. Wives are too often the arbiters of the family mode of living. They measure the husband's purse string and then begin to try how much it will stretch, and they pull and pull until one of two things happens, the string breaks or they die from the overstrained effort. We all try to see how much we can swell out. Our grounds must be just a little broader than we can till; our house a little bigger than we can fill; our furniture a little better than we can buy; and to make up the deficit a corresponding sacrifice must be made somewhere, and that is generally a sacrifice of happiness and health, to what? To Mrs. Grundy.

In these times, when retrenchment is necessitated by stringent circumstance, and many are compelled to abandon high hopes and accept the inevitable, one long step down is far easier to take than a succession of short ones; and hence they will prove themselves wise, who, finding that they will be less conspicious in their down stepping by reason of the multitude of those to whom attention therefor is called, shall strike a basis commensurate with their minimum means rather than with their maximum expectations. When one arrives at that stage in which one's own comfort and happiness is consulted, rather than somebody's opinion thereof; when cosy homes, that will not entail a life-time of drudgery to superintend, shall become the successors of great establishments that serve only to butt a criticism against, then shall the mistresses thereof find time to devote themselves to something more elevating than doing a menial's work at society's bidding, and wearing out their strength and life in a fretful unrest lest the conventionalities of the arbitrary circle in which they cannot afford to move, be violated forsooth, and they become outcasts therefrom. Perhaps the present crisis in finance is one of the inscrutable means to the attainment of some such happy results as this, and if so, ambitious aspirations should be gladly sacrificed to homely comfort and contentment, if, along with, and greater than all else, the restoration to health of a large class of the community is thereby effected. There is a growing sentiment at this time in favor of contractions of all sort, and the wisdom which common sense suggests and which reason approves, and none but the silly condemn, is the abandonment of the fictitious for the real, and a determination on the part of all, let come what may, to live within their means, however humble a position it may place them in. Such a movement, should it become general, will not only save us from pecuniary embarrassments, but go far towards restoring to their normal health and happiness, the jaded and faded who have become such by endeavoring to seem what they are not.

